# The Limitations of Medical Opinion

**Published:** 1915-09-25

**Authors:** 


### The Hospital

The Workers' Newspaper of Administrative Medicine and Institutional Life, Administration, National Insurance and Health.

No. 1528, Vol. LVIII. SATURDAY, SEPTEMBER 25, 1915. PRICE ONE PENNY.

### THE LIMITATIONS OF MEDICAL OPINION.

The truth of the aphorism that "art is long" has been abundantly illustrated in many fields, and even in an age in which short-cuts are popular the doctrine compels recognition. Probably in no department of activity is this teaching more forcibly demonstrated than in the study of medicine. The records of the past are crowded with beliefs and opinions and practices, all commanding in their day, but now known only to the few whose inclination leads them to rummage amongst the forgotten. The explanation is, of course, that incomplete or inaccurate observation was followed by hasty and ill-founded conclusions, and that time has mocked at the process. To censure the past is easy; it is less easy to gather its lessons, and the record of the present will doubtless supply abundant instances to show that in the direction here indicated history repeats itself. There are at least two reasons which explain why ill-founded judgments are specially prone to occur in medical opinion and practice. One of these is that disease in the individual is an event which cannot await the process of severe and impartial experiment; the other, that the materials for an established conclusion are often extremely difficult to collect.

The desire of a sick man is to get well, and he is ready to use all the methods likely to promote this purpose. Much the same attitude is true of his medical adviser. Neither one nor the other is primarily concerned with the truth or falsity of any particular pathological or therapeutic truth. Hence the clinical conditions do not invite or even admit the institution in anything like a rigorous fashion of the experimental method. So long as the patient receives benefit he is content, and, very naturally, he has little inclination to inquire which, if any, of the means employed must be credited with curative value. Very similar conditions are imposed on the medical practitioner, whose first duty is to help his patient and not closely to analyse the precise contribution made by each of the agencies employed to this end. Some conclusions on this latter point he will doubtless form in his own mind, and he may readily fail to remember that these lack the essential conditions of scientific proof. His experiences, when published, must attract the attention of others, who will feel more or less compelled to imitate a method for which value is claimed. Thus the teaching may affect a wide circle, and it may be long before conditions arrive which test the proposition in a satisfactory and conclusive manner. Yet, in the end, it may be shown that the opinion and the practice founded on it were destitute of all firm basis, and they come, perhaps after a relatively long life, to be gathered into the dust-heap and to be forgotten. Doubtless, as an exercise in logical reasoning, the process merits condemnation, but viewed in its practical relations it is not without defence. The problem, we repeat, is to get the sick man well, and both he and his adviser are driven to anything which, so long as it will not do harm, offers a chance in this direction. It is, of course, very desirable that we should have certain knowledge, but the actual conditions often forbid us to wait for this, and a reasonable presumption is all that is possible. To quarrel with circumstances which cannot be altered is a mere peevish waste of time, and duty and common-sense tell us to make the best of them. In the issue here under discussion the erection of conditions such as are demanded by a strict scientific inquiry are not possible, and this fact offers a partial explanation of the accumulation of discarded views and forgotten doctrines which mark the history of medical progress. It is necessary to remember all the circumstances, and this both in judging the past and in seeking guidance for the future. On the latter point it is sufficient to say that the frequency of error in the past is a warning for the present, and the discovery of excuses does not carry the suggestion that inaccurate observation and hurried judgments are mere venial offences. On the contrary, they are extremely serious, and our aim is not to find excuses for them but to show how much they have contributed, and continue to contribute, to an instability of opinion which is sometimes made a matter of reproach to the profession. The circumstances make them hard to avoid; all the more reason that effort should be made to resist them.

Another condition of medical work which makes judgment difficult and even experience fallacious is provided by the obstacles which exist to the collection of all the facts. Rare and exceptional pathological events sometimes produce symptoms which ordinarily have some simple causation, and the uncommon explanation can only be known to the few individuals who have had experience of it. It is simply impossible for any practitioner, however studious be his habits, to be familiar with all the odd causes that have been recorded, and yet at any moment he may meet a parallel instance. Again, in many forms of chronic disease the drama is a very long-drawn-out one, and one and the same practitioner by no means sees all the acts. He thus fails to secure for his later guidance the help which a fuller experience would have given him. What he has to deal with is often merely a chapter in a long story of which he may see neither the beginning nor the end. Thus the absence of a complete reading leaves some degree of uncertainty in his mind, and ill prepares him for a definite and secure opinion when conditions apparently similar are again forced on his view. The public, not unnaturally, calls for dogmatic confidence—and sometimes gets it—when in the very nature of things such confidence is not possible. These and related circumstances are among the hindrances to certain medical knowledge. They are well known to the profession, though little appreciated outside. Infallibility is no claim of medicine, and if there is greater difference of opinion here than elsewhere this means, not that doctors are exceptionally stupid, but that the conditions of their work are exceptionally difficult.

